# Strategy effects and value directed recall of sub- and supra-span word lists

**DOI:** 10.3758/s13421-025-01828-4

**Published:** 2026-05-19

**Authors:** Harini J. Babu, Tiffany K. Jantz, Kathy Y. Xie, Patricia A. Reuter-Lorenz

**Affiliations:** Department of Psychology, University of Michigan, Ann Arbor, MI USA

**Keywords:** Value-directed recall, Working memory, Prioritization, Metamemory

## Abstract

Value cues can promote selective processing of words in lists of 12 or more items leading to better recall of memoranda associated with higher value. The current study examined whether value cues can also benefit memory for words studied in shorter lists, including those within the normative range of verbal working memory span (~four items). Across four experiments, participants studied words randomly paired with different point values in lists of three to 12 items, followed by immediate free recall. When list length varied randomly and participants could not anticipate memory set size, value-directed effects were evident for all list lengths: Words of higher-value were better recalled than lower-value words, even for lists lengths 3 and 4, demonstrating value-directed memory (VDM) effects within the canonical parameters of working memory. In contrast, with advanced knowledge of list length, value effects emerged only for lists of five or more words and overall recall was better for lower value words than when list length was unexpected, especially for shorter lists. Serial position and output order analyses corroborated these results. The current VDM effects, found in-person, and replicated with online testing, extend prior evidence indicating prioritization based on value within the parameters of working memory. The results also underscore the importance of metamemory in value-based effects: When anticipating word lists with relatively few items (three or four words), value cues can be ignored in favor of more efficient memory strategies, which results in discontinuities in VDM effects for shorter versus longer lists.

**Supplementary Information:**

The online version contains supplementary material available at 10.3758/s13421-025-01828-4.

## Introduction

Given the limits of human memory, it is often necessary to selectively prioritize a subset of information that is important or valuable for achieving our current goals. Understanding how such prioritization is achieved and the constraints on selective remembering can shed light on the processes that optimize memory. In the laboratory, *value-directed memory* (VDM) paradigms aim to imbue “importance” or priority in memoranda by assigning different point values to long lists of items. Memory is then tested to determine whether an item’s memorability was influenced by its associated value. Numerous studies using this paradigm have found that memory is superior for higher than lower-valued items demonstrating that memory can be prioritized based on assigned value (for a review, see Knowlton & Castel, 2022). Thus far, VDM effects of this sort have been documented for recall and recognition of lists typically consisting of 12 or more words (Castel et al., 2002, 2007, 2009; Cohen et al., 2017; Friedman & Castel, 2011; Hennessee et al., 2017). Here we investigate whether value can also influence immediate memory for words in shorter lists including those within the normative parameters of verbal working memory capacity (i.e., short-term memory span),^1^ typically considered to be four items or less (Barrouillet et al., 2021; Cowan, 2001, 2010; Halford et al., 2007).

Prioritization effects have been widely demonstrated in working memory and indicate that one member of a small set of memoranda can be preferentially retained for immediate recall or recognition (e.g., Myers et al., 2017). However, the methods used to investigate prioritization in working memory differ substantially from those used to study value-directed effects in long-term memory. In a typical VDM task, each word in a supra-span list is presented with a different point value and participants are asked to remember as many words as possible, while maximizing the points earned. Point values vary randomly, requiring that participants dynamically update their priority list, and evaluate which words to promote as each successive item is presented. The memory benefits of higher value are robust for words in lists ranging from 10 to 90 items; they are observed in immediate and delayed recall (Castel et al., 2002, 2007; 2009; Cohen et al., 2017; Friedman & Castel, 2011; Hennessee et al., 2017), and in anticipated and surprise recognition tasks (Castel et al., 2007; Elliot & Brewer, 2019; Elliot et al., 2020).

In contrast, prioritization effects in working memory have been typically studied in tasks using colored shapes, numbers, or letters, where pre-cues or retro-cues specify a location to prioritize among a small set (i.e., four items) of simultaneously (Atkinson et al., 2022; Allen & Ueno, 2018, Experiment 1) or sequentially presented stimuli (Hitch et al., 2018; Hu et al., 2014). Other studies have examined prioritization of a specific spatial position or serial position in an upcoming sequence of items (i.e., ranging from four to seven items; Atkinson et al., 2021; Hu et al., 2016; for a review, see Allen et al., 2024). Concurrent color cues have also been used to distinguish the high-value item from the others in the memory set (e.g., one red among all black items; Sandry & Ricker, 2020). Memory for the single prioritized item is rewarded with more points relative to all other items, which are typically assigned equal value, resulting in superior memory for cued items compared to uncued items, or compared to conditions in which all items have equal value (Atkinson et al., 2022, 2024; Allen et al., 2021, 2024; Hu et al., 2023; Sandry et al., 2014, 2020). While prioritization effects have been demonstrated most frequently in visual working memory using cued recall or recognition (Atkinson et al., 2018; Hu et al., 2016; Jeanneret et al., 2023), they are also evident for working memory in the auditory (Atkinson et al., 2021), tactile (Roe et al., 2024), and olfactory modalities (Johnson & Allen, 2023).

Benefits for prioritized items in working memory are hypothesized to result from the preferential allocation of attentional resources to the cued items, relative to uncued items, and relative to a neutral allocation policy in conditions where all items have equal value (Atkinson et al., 2019; Hitch et al., 2018). Prioritized access to the focus of attention leads to better memory over the short and longer term (Jeanneret et al., 2023; Sandry et al., 2020; Sandry & Ricker, 2020; however, see Atkinson et al., 2024), and to faster response times (Sandry et al., 2014; Sandry & Ricker, 2020). When value is cued within or prior to the display, preferential rehearsal could also underlie superior memory for the high-value item (Stefanidi et al., 2018). However, preferential rehearsal as an alternative and general mechanism for prioritization effects in working memory has been challenged because these effects are evident despite concurrent articulatory suppression (Allen et al., 2021; Atkinson et al., 2018, 2019) and with non-verbal memoranda, such as non-sense shapes, symbols, olfactory and tactile stimuli (Adcock et al., 2006; Roe et al., 2024; Sandry & Ricker, 2020).

In long-term memory paradigms, by contrast, VDM effects are hypothesized to depend on the strategic promotion of high-value words by engaging in more elaborative and deeper encoding compared to those of lower value (Knowlton & Castel, 2022). In line with this interpretation, measurement of pupil diameter suggests that participants engage more resources and greater effort when processing high versus low-value words (Ariel & Castel, 2014; Bergmann et al., 2019). Additionally, neuroimaging has shown that brain regions commonly associated with semantic processing are more active when encoding high-value words than low-value words (Cohen et al., 2014). Furthermore, participants’ knowledge about task parameters and thoughts about their own memory abilities (i.e., metamemory) can impact strategy use and the magnitude of VDM effects (Castel et al., 2012; Castel et al., 2015), consistent with the deliberative engagement of memory control processes. For example, VDM effects increase after the first few lists suggesting that participants learn to maximize performance as they become familiar with the task parameters and their own memory limitations (Castel et al., 2002; Castel et al., 2007; Castel et al., 2009; McGillivray & Castel, 2011). Moreover, participants remember fewer high-value items when they anticipate recognition testing rather than recall (Middlebrooks et al., 2017). These findings indicate that participants’ strategic use of value is influenced by their knowledge of task conditions and judgements about their memory abilities.

Nevertheless, some benefits of value have been attributed to more automatic, reward-based dopaminergic mechanisms thought to operate during long-term memory consolidation (Adcock et al., 2006; Bowen et al., 2020; for a review, see Knowlton & Castel, 2022). Although reward motivated learning of this kind is typically evident after long (24-hour) delays, there are indications that even with same-session testing, VDM effects on verbal recognition may stem in part from automatic effects of value. For example, within a single experimental session, Hennessee et al. (2019) report that value-directed effects persisted even when participants were informed via forget instructions that value cued items were no longer relevant, suggesting that memory enhancement occurred effortlessly. To the extent that value can enhance memory automatically, VDM effects should be somewhat resilient to effects of strategy.

While VDM effects for lists of verbal (word) stimuli are well established within the parameters of long-term memory, little is known about how dynamically presented value cues would affect working memory for word lists. That is, would VDM effects emerge for words paired with value cues when presented and tested within the canonical parameters of working memory (i.e., immediate short-term recall or recognition of lists of approximately four or fewer items, see Barrouillet et al., 2021; Cowan, 2001)? While evidence for prioritization effects in working memory might favor similar outcomes with words (Allen & Ueno, 2018; Atkinson et al., 2021, 2024; Hu et al., 2014; Sandry et al., 2020), further investigation is necessary to determine the generalizability of these effects given that VDM tasks in long-term memory use different methods and are hypothesized to harness deeper, elaborative encoding strategies characteristic of long-term memory processing (e.g., Cohen et al., 2014). Thus, the mnemonic control processes thought to give rise to VDM effects for lengthy word lists may differ from those that underlie the value-based prioritization effects in working memory reported thus far. Therefore, it remains an empirical question as to whether VDM effects can be documented for short lists of sequentially presented words paired with dynamically changing value cues and recalled from working memory.

According to embedded-process or unitary store models of memory, VDM effects should also be expected for list-lengths at or near working memory span. This view posits that human memory comprises a single, unitary system in which the contents of working memory entail the temporary, capacity-limited activation of long-term representations (Cowan, 1999; Jonides et al., 2008; Nairne, 2002; Oberauer, 2002). Conceivably then, the control processes that give rise to value-directed effects on immediate recall for supra-span lists could also be applied to lists within the capacity of working memory. Indeed, memory phenomena previously thought unique to long-term memory, such as depth-of-processing (Flegal & Reuter-Lorenz, 2014; Rose & Craik, 2012), directed forgetting (Festini & Reuter-Lorenz, 2013; Oberauer, 2001), proactive interference (Atkins et al., 2011; Keppel & Underwood, 1962), and false memories (Dimsdale-Zucker et al., 2019; Flegal et al., 2010; Olszewska, et al., 2015) have also been observed in tasks characterized by canonical working memory parameters, suggesting that similar memory principles apply to sub- and supra-span lists and across short and long delays. Multistore models of memory, on the other hand, argue that working memory and long-term memory are separable systems, subject to different memory operations and processing constraints (Baddeley, 2012; Logie, 2011; for a review of unitary and multistore models of memory, see Adams et al., 2018). As such, it is plausible that value-directed memory effects for words, and the elaborative encoding processes hypothesized to support them, are unique to long-term memory and would not be evident within the canonical parameters of a working memory task.

Here, we investigate these possibilities by measuring VDM effects for words comprising list lengths ranging from three to 12 items. In the first experiment, conducted in person, participants performed a VDM task with words presented sequentially in lists of three, six, nine, or 12 items. Because lists length was randomized, participants could not anticipate the memory load for any given trial. To assess potential strategic contributions to these value-based effects, Experiment 2 used the same in-person procedure, but list length was blocked and participants were informed about list length at the start of each block (see Grenfell-Essam & Ward, 2012, for a similar foreknowledge manipulation). Experiments 3 and 4 replicated the effects in Experiments 1 and 2 respectively, when participant samples were tested online and with slight variations in the VDM methods, including list lengths ranging from four to 11 items.

## Experiment 1

### Method

#### Participants

Fifty-three young-adult participants completed the study.^2^ A sample size in this range was based on prior studies of value-directed remembering (e.g., Castel et al., 2009; Castel et al., 2011). Participants were recruited through the University of Michigan’s Introductory Psychology Subject Pool. Participants received course credit as compensation. To increase point value salience, participants received a $5.00 performance-based bonus if they earned at least 250 points (out of 2,340) over the course of the experiment (Castel et al., 2009; see *Procedure*). Participants completed the study in person in a research laboratory. Informed consent was obtained prior to participation and all experimental procedures were approved by the University of Michigan’s Institutional Review Board. All participants were treated within the ethical guidelines of the American Psychological Association.

Seven participants were excluded in data analysis for being younger than 18 years, being non-native English speakers, due to computer or experimenter error, or for earning fewer than 250 points (indicating insufficient effort; see Appendix A in the Supplemental Materials for “points-earned” data for all experiments). The final sample included 46 young adults (18–22 years, *M*_*age*_ = 18.89 years; 69% male;^3^ see Appendix B in the Supplemental Materials for race and ethnicity). A post hoc power analysis performed using the *simr* package (v. 1.0.7; Green & MacLeod, 2016), indicated that this sample size had 100% power to detect our effects of interest.

#### Materials

Using the MRC database (https://websites.psychology.uwa.edu.au/school/MRCDatabase/uwa_mrc.htm), 381 words were selected for the value-directed memory (VDM) task with the following characteristics: three to eight letters in length, one to three syllables, familiarity of 400–640, concreteness rating of 300–600, and Kucera and Francis written frequency of 10–150. Words were randomly sorted into three practice lists of three, six, and 12 words and 48 experimental lists of three, six, nine, or 12 words. There were 12 experimental trials of each list length. Each word was assigned a value from 1 to 12.

#### Procedure

**Fig. 1:**
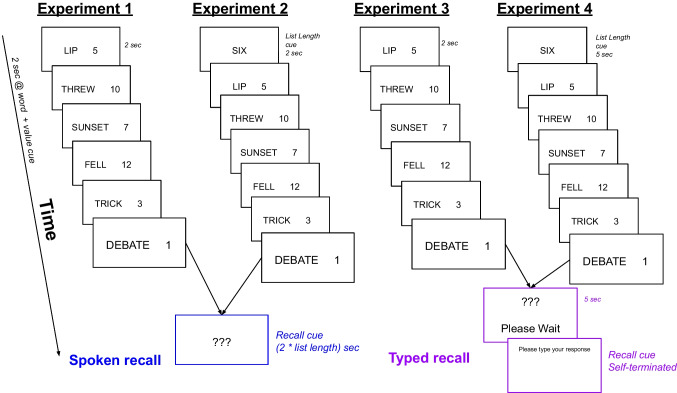
The Value-directed Immediate-recall Task. *Note. *Participants studied lists ranging from 3 to 12 words. Words were associated with low (1–4), medium (5–8), or high (9–12) values. In Experiments 1 and 3, list length varied randomly and was unknown to the participants in advance. In Experiments 2 and 4, list length was blocked and participants were informed of the length at the start of each block. Following presentation of the word-value pairs, participants were prompted to verbally recall the studied words (Experiments 1 and 2) or were prompted to type their responses (Experiments 3 and 4).

Each value-directed memory (VDM) trial began with the presentation of a fixation star in the center of the screen (0.5 s), followed by the presentation of a word and its associated point value (see Fig. 1). As in Castel et al. (2009), each word and point value appeared in the center of the screen for 2 s. Word-point pairs were presented sequentially with an inter-stimulus interval (ISI) of 0.5 s. The last word of each list was followed by a recall cue, three question marks “? ? ?”, which prompted participants to say aloud all the words they could remember from the current trial. The recall cue duration varied based on list length with 2 s given for each word (e.g., 6 s for a three-word list). Following Castel et al. (2009), participants were instructed to try to maximize the number of points they earned, while also trying to recall as many words as possible from each list. Appendix Table A1 in the Supplemental Materials displays summary statistics for points earned across all experiments. Participants’ correct and phonetically similar^4^ verbal responses were recorded by a research assistant who was present in the room.

Before beginning the VDM task, participants completed three practice trials consisting of three-, six-, and 12-word lists in a randomized order. The main task consisted of 48 VDM trials. There were 12 trials for each of the four list lengths (LL), LL3, LL6, LL9, and LL12 words. List length varied pseudorandomly such that any particular list length did not appear more than twice in a row and list order was randomized. Participants did not know and could not anticipate how many words were in a given trial until the recall cue appeared. As in previous research (Castel et al., 2002; Castel et al., 2009), each word was randomly paired with a point value ranging from 1 to 12 and no point value was repeated within a trial. For lists with fewer than 12 words, not all point values could be represented. To maintain balance across list-lengths, point values were grouped into three levels: low (1–4 points), medium (5–8 points), and high (9–12 points). Point groups (high, medium, low) were equally represented both within each trial and across all 48 trials. All point values were equally represented across the 48 trials and across participants, word-point range pairings were randomized such that no word was consistently associated with a particular value. The experiment was implemented using E-Prime 2.0 software (Psychology Software Tools, Inc., 2016).

Following the immediate-recall task, participants completed a 2-min unfilled break during which they were informed of their total points earned and were awarded their performance-based bonus if they met the 250-point threshold. After the break, participants completed a surprise, untimed delayed recall task where they recalled as many words as possible from the prior phase of the experiment (for Experiments 1 and 2 only). However, the few numbers of words recalled in this task (mean = 5.6%, range = 6–54) precluded any informative analyses based on list length. Therefore, this task will not be further discussed. Lastly, participants completed a brief, open-ended exit survey to assess reports of strategy use. Appendix C in the Supplemental Materials contains the surveys used for each experiment, and a brief summary relevant to strategy use.

#### Data analysis

For this and the following experiments, we calculated the proportion of words recalled by dividing the number of words correctly recalled in each point value group by the total number of words in the list with that point value. For example, suppose a participant correctly recalled one high-point value word within a nine-word list trial, and there were three occurrences of high-point value items. In that case, the proportion of high-point value words in the trial is 0.33 (1/3)^5^.

To further characterize the effects of value on lists of different lengths, we calculated the proportion of words recalled as a function of serial position (SP) and output position (OP) for each list length.^6^ The proportion recalled by SP was computed by dividing the number of correctly recalled words in each point value category for each specific SP and within each list length by the total number of words presented in the same point value category at the SP and list length. For example, across all three-item lists, a participant saw four high-point value words at SP 1 and of these words, they correctly recalled two. Therefore, the proportion of high point value items recalled at SP 1 for three-item lists would be 2/4 = 0.5.

The proportion recalled by OP was calculated by dividing the number of correctly recalled words in each point value category at a given OP and list length by the total number of correctly recalled words at the same OP and list length. For example, within the set of 12-item lists, if a participant correctly recalled 10 words at OP 1, and two of those were high-point value words, then the proportion of high-point value items recalled at OP 1 would be 2/10 = 0.2. This approach for calculating recall proportions for high-, medium- and low-value words across SP, OP, and list lengths was applied consistently across all experiments.

For all experiments, statistical analyses were conducted using R (R Core Team, 2013 version 2023.12.1). We conducted repeated measures analyses of variance (ANOVA) using the *ez* (version 4.4-0; Lawrence, 2016) and *afex* packages (version 1.3-0; Singmann et al., 2023). Greenhouse-Geisser corrections are applied when assumptions of sphericity are violated. Contrasts examining significant main effects and interactions were conducted using the *emmeans* package (version 1.10.0; Lenth, 2024). *P-*value significance cutoffs are adjusted using the Holm-Bonferroni correction for family wise error rates (Holm, 1979). Null or nonsignificant contrasts were further assessed by calculating Bayes factors (*BF*) using the *BayesFactor* package (version 0.9.12-4.7; Morey & Rouder, 2024). Per standard interpretations, *BF* values below 1 suggest evidence for the null hypothesis, BF values close to 1 suggest the data are insensitive to the null or alternative hypothesis, and BF values substantially larger than 1 suggest evidence for the alternative hypothesis (Dienes, 2014; Andraszewicz et al., 2015).

All repeated-measures ANOVAs included high, medium, and low point value words. Although word recall in the medium point value condition most often fell between the high and low-value conditions, we characterized the presence or absence of a VDM effect for each list length based on whether recall for high- and low-value words differed reliably.

### Results

**Fig. 2:**
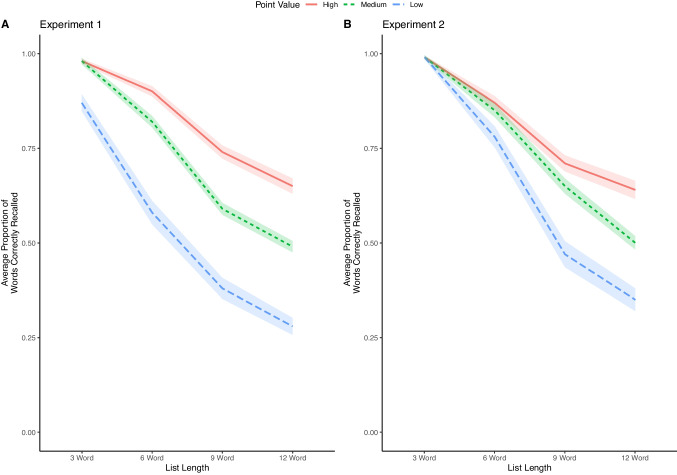
Average Proportion of Words Correctly Recalled by List Length and Point Value Category in Experiments 1 and 2. *Note.* In Experiment 1, participants had no foreknowledge of list length; in Experiment 2 list length was known in advance. Proportion of words recalled by list length (3, 6, 9, and 12 words) and point value: high (red, solid line), medium (green, dotted line) and low (blue, dashed line) in Experiment 1 (**A**) and Experiment 2 (**B**). Shaded areas around each line represent the standard error.

A 3 × 4 ANOVA compared the proportion of words recalled, where point category (low, medium, and high) and list length (LL3, LL6, LL9, and LL12) were within-subject factors (see Fig. 2A). The omnibus ANOVA revealed a main effect of point value, *F*(1.15, 51.56) = 75.11, *p* < .001, η^2^_p_= 0.63, a main effect of list length, *F*(2.40, 108.19) = 816.01, *p* < .001, η^2^_p_= 0.95, and a significant interaction between list length and point value on proportion of words recalled, *F*(4.54, 204.34) = 25.20, *p* < .001, η^2^_p_= 0.36.

Contrasts examining the main effect of value revealed that participants recalled larger proportions of high- than medium-value words, *t*(45) = 8.25, *p* < .001, d = 1.36, medium- than low-value words, *t*(45) = 7.91, *p* < .001, d = 1.59, and high-than low-value words, *t*(45) = 9.14, *p *< .001, d = 2.33. Contrasts examining the main effect of list length replicate previous reports (e.g., Ward et al., 2010) and revealed that participants also recalled smaller proportions of words from LL12 compared to LL9, *t*(45) = 12.57, *p* < .001, d = 1.38, from LL9 versus LL6, *t*(45) = 22.01, *p* < .001, d = 2.64, and from LL6 compared to LL3, *t*(45) = 17.72, *p* < .001, d = 2.60.

These main effects are qualified by a point value by list length interaction in which the effect of point value varies by list length. As seen in Fig. 2A, the recall difference between point values within the LL3 is smaller than other memory sets. Regardless, recall for high-point value words was significantly greater than those with low point value across all list lengths, and for all but the shortest list length recall for medium-value items fell between high and low point values. For LL12, the typical list length used in previous value-directed memory experiments, participants recalled more high-value than low-value words, with recall for medium-value words falling in between. Similar patterns emerged for LL9 and LL6 (for all other pairwise comparisons see Table 1).

**Table 1:** Comparisons of Point Value Effects on Proportion of Words Recalled for each List Length in Experiments 1 and 2

	Experiment 1			Experiment 2		
	High vs. Medium	High vs. Low	Medium vs. Low	High vs. Medium	High vs. Low	Medium vs. Low
**3-word**	*BF* = 0.24	4.52 *p* = .001 *d* = 0.1	4.44 *p* = .002 *d* = 0.1	*BF* = 0.38	*BF* = 0.29	*BF* = 0.23
**6-word**	4.91 *p <* .001 *d* = 0.08	8.54 *p <* .001 *d* = 0.3	6.78 *p <* .001 *d* =0 .22	*BF =* 0.30	3.06 *p* = .01 *d* = 0.08	2.69 *p* = .03 *d* = 0.06
**9-word**	7.45 *p <* .001 *d* = 0.14	8.9 *p <* .001 *d* = 0.33	6.76 *p <* .001 *d* = 0.19	2.89 *p =* .02 *d* = 0.05	5.23 *p <* .001 *d* = 0.21	4.94 *p <* .001 *d* = 0.16
**12-word**	6.61 *p <* .001 *d* = 0.14	9.85 *p <* .001 *d* = 0.34	8.99 *p <*.001 *d* = 0.19	5.6 *p <* .001 *d* = 0.13	6.46 *p <* .001 *d* = 0.27	5.58 *p <* .001 *d* = 0.14

*Note.* Contrasts are presented for Experiment 1 (without foreknowledge of list length) and Experiment 2 (with foreknowledge of list length). Significant contrasts display a t-statistic (Degrees of Freedom: Experiment 1 = 45; Experiment 2 = 43), the associated Bonferroni-corrected p-value and effect size (Cohen’s d). Strength of evidence for the null hypothesis for non-significant comparisons was assessed with Bayes Factors (*BF*). *BF* values < 1 indicate greater support for the null hypothesis; *BF* values > 1 favor the alternative hypothesis.

Critically, for LL3, which presumably does not exceed average working memory capacity, participants also recalled significantly more high-value words than low-value words, and more medium-value than low-value words, even at this very short list length. There was no difference in recall rates between medium and high-value words likely because performance was near ceiling.

#### Effects of value on serial positions and output order

**Fig. 3:**
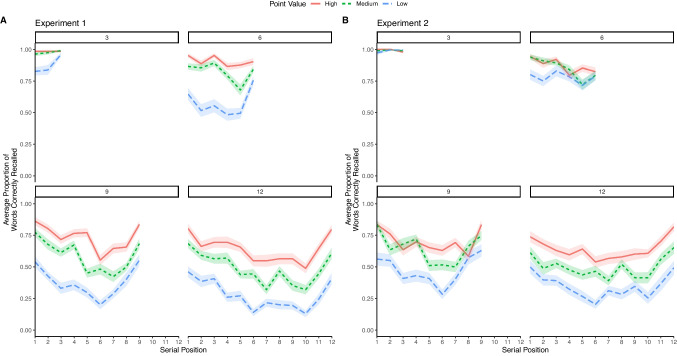
Average Proportion of Words Recalled by Serial Position, List Length, and Point Value Category in Experiments 1 and 2. *Note. *Proportion of words recalled by serial position (1–12), list length (3, 6, 9, and 12 words) and point value: high (red, solid line), medium (green, dotted line) and low (blue, dashed line) in Experiment 1 (**A**) and Experiment 2 (**B**). Shaded areas around each line represent the standard error.

To assess how value influences SP within each list, and whether these effects vary by list length, the proportion of words recalled was plotted as a function of SP for each list length. As can be seen from Fig. 3A, while primacy and recency effects are most evident for LL6–12, there is a pervasive tendency to recall high and medium-value words relative to low-value words across all SPs. The most recent position for LL3 is an exception in that low-value words are most likely to be recalled from this position, an effect that is somewhat apparent for LL6, whereas the effects of value are more equal across SPs for LL9 and LL12.

**Fig. 4:**
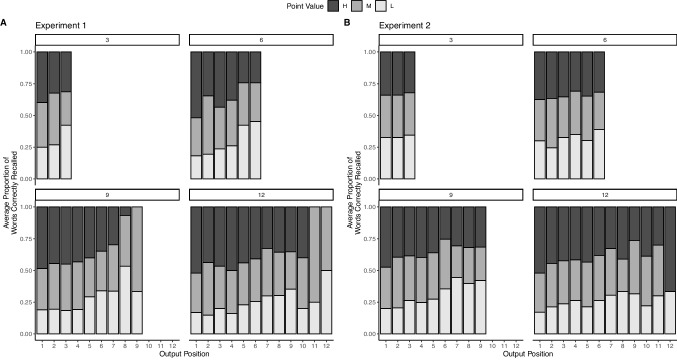
Average Proportion of Words Recalled by Output Position, List Length, and Point Value Category in Experiments 1 and 2.* Note.* Proportion of words recalled by output position (1–12), list length (3, 6, 9, and 12 words) and point value: high (dark gray), medium (gray) and low (light gray) in Experiment 1 (**A**) and Experiment 2 (**B**)

To assess how value affects OP within each list and if they vary across list length, the proportion of words recalled for each value category was plotted as a function of OP for each list length. As can be seen from Fig. 4A, high- and medium-value words are the most likely to be generated as the first and second response for all list lengths, whereas the proportion of low-value words recalled generally increases with increasing OP.

### Discussion

The results from Experiment 1 indicate value-directed memory effects on immediate recall from lists of all lengths, including those near (LL6) or within (LL3) canonical estimates of working memory span. Regardless of list length, recall was greater for high-value words and their output was prioritized relative to low-value words. Words associated with intermediate values were also recalled more than low-value words for all list lengths, and less than high-value words except for LL3 where recall for high- and medium-value words did not differ, likely due to ceiling level performance. Better recall of high- and medium-value words compared to low-value words was also evident across all serial positions for all list lengths, except for LL3, where words in the most recent serial position were approximately at ceiling and minimally affected by value. Overall, these findings extend the evidence for prioritization effects in working memory to lists of words associated with dynamically changing point values. The results are consistent with the possibility that processes that give rise to VDM effects with longer lists can also be brought to bear on encoding items in shorter lists. Alternatively, different prioritization strategies could be used for different list lengths, such that higher value words are maintained in the focus of attention for shorter lists, whereas deeper, more elaborative encoding is engaged for higher value words as list length exceeds capacity. Lastly, to the extent that value effects are automatic, they could emerge for all list lengths with minimal contributions from strategic processing.

A key design feature in the first experiment is that participants could not anticipate the number of words to be retained in a given trial. This makes it unlikely that participants used different strategies based on list length because they had no knowledge of how many words they would be required to retain until the recall prompt. Nevertheless, if VDM effects for short versus long lists were achieved by different strategies when list length was unknown, then these effects should be replicated and may be even more pronounced when participants have advanced knowledge of list length. Furthermore, to the extent that the effects of value are automatic, they should persist even with foreknowledge of list length. Alternatively, participants might opt to refrain from strategies such as deeper elaborative encoding, if more efficient options like rote or maintenance rehearsal would suffice, especially for shorter lists. Doing so could reduce value effects for shorter lists when list length was known in advance. The next experiment tested these hypotheses by informing participants of the forthcoming list length and presenting each list length in separate blocks.

## Experiment 2

### Method

#### Participants

Forty-six participants from the University of Michigan’s Introductory Psychology Subject Pool completed the study-tasks in a research laboratory. As in Experiment 1, informed consent was obtained prior to participation and all experimental procedures were approved by the University of Michigan’s Institutional Review Board. Participants received course credit for their participation. As in Experiment 1, participants received a $5.00 performance-based bonus at the end of the experiment if they earned more than 250 points (out of 2,340). Two participants were excluded from the analysis for earning less than 250. The final sample size included forty-four young adults (18–22 years, *M*_*age*_ = 18.86 years; 45% male; see Appendix B in the Supplemental Materials for race and ethnicity).

#### Materials

Materials were identical to those used in Experiment 1.

#### Procedure

The procedure was identical to Experiment 1 except that trials were blocked by list length and participants were informed of the list lengths for the proceeding block of trials (see Fig. 1). For example, prior to the onset of a three-item block, participants saw a screen (2 s) with the word ‘THREE’ denoting the length of the list. There were four blocks containing 12 trials each. Blocks of trials varied pseudorandomly across participants and were never arranged in ascending or descending order by list length. As in Experiment 1, participants were instructed to recall as many words as possible while trying to maximize the number of points earned (Appendix A in the Supplemental Materials).

### Results

We examined the effect of point value on proportion words recalled for each list length by conducting a 3 × 4 repeated-measures ANOVA, where point category (low, medium, and high) and list length (3, 6, 9, and 12) were within-subject factors. Summary statistics are displayed in Fig. 2B. The omnibus ANOVA revealed a main effect of point value, *F*(1.23, 52.94) = 31.33, *p* < .001, η^2^_p_= 0.42, a main effect of list length, *F*(2.49, 107.22) = 545.65, *p* < .001, η^2^_p_= 0.93, and a significant interaction between list length and point value on proportion of words recalled, *F*(3.27, 140.80) = 19.11, *p* < .001, η^2^_p_= 0.31.

As in Experiment 1, planned contrasts revealed a value-directed memory effect as indicated by greater proportional recall of high-value words than medium-value words, *t*(43) = 4.77, *p* = .001, d = 0.66; of medium-value words compared with low-value words, *t*(43) = 5.18, *p *< .001, d = 0.88, likewise for high-value words compared with low-value words, *t*(43) = 5.96, *p *< .001, d = 1.35. Once again, planned contrasts investigating the main effect of list length revealed that as list length decreased, participants recalled higher proportions of studied words; participants recalled a smaller proportion of words from LL12 versus LL9, *t*(43) = 10.27, *p* < .001, d = 1.09, LL9 versus LL6, *t*(43) = 19.83, *p* < .001, d = 2.06, and from LL6 versus LL3, *t*(43) = 9.94, *p* <.001, d = 2.00.

Of importance, these main effects were qualified by a significant list length by point value interaction. For LL3, there were no differences among any of the point values, whereas all other list lengths showed the expected VDM pattern especially when comparing recall for high versus low value words (see Table 1 for all comparisons).

#### Effects of value on serial positions and output order

Once again, SP and OP were calculated as a function of value and plotted for each list length. Figure 3B illustrates that there is no effect of value on any SP for LL3, as would be expected given the lack of overall value effects on recall for this list length. Likewise for LL6 and LL9, value effects are still evident but attenuated except for the most recent SP for LL9. Finally, for LL12, value effects are evident across all SPs.

With respect to OP plotted in Fig. 4B, LL3 shows no effect of value for any output position. For LL6, high- and medium-value words are more likely to be output first or second, whereas the subsequent OPs are insensitive to value, except for OP6 where low-value words are most likely. For LL9 and LL12, high- and medium-value words are most likely to be recalled in the first half of responses generated, whereas recall of low-value words increases in the latter half of each response set.

### Comparison of Experiments 1 and 2

Results of Experiment 2 indicate that immediate recall is virtually perfect regardless of associated point values for LL3, which is likely within working memory capacity. Knowing the list length in advance, participants may have been less inclined to attend to, or use, the value cues to recall LL3, whereas VDM effects were still evident for LL6 or more. This pattern suggests that when given foreknowledge, participants may have adopted different memory strategies depending on list length, as opposed to adopting more uniform strategies when memory load could not be anticipated in Experiment 1. To assess these differences statistically, Experiments 1 and 2 were compared directly to quantify the effects of foreknowledge.

We conducted a 2 × 3 × 4 mixed-design ANOVA comparing proportion of words recalled, with experiment (1 vs. 2) as the between-subjects factor and value (high, medium, low) and list length (3, 6, 9, 12) as within-subject factors. The omnibus test revealed similar findings as the results of each individual experiment: there were main effects of point value, *F*(1.18, 103.86) = 103.94, *p* < .001, η^2^_p_= 0.54, and list length, *F*(2.61, 230.08) = 1,305.39, *p* < .001, η^2^_p_= 0.94, and a significant interaction *F*(4.01, 353.31) = 39.04, *p* < .001, η^2^_p_= 0.31. Of particular interest are the differences between the two experiments: A main effect of experiment, *F*(1, 88) = 10.84, *p* = .001, η^2^_p_= 0.11, suggests significantly better overall memory performance with advance knowledge of list length (Experiment 2, *M* = 0.73, *SE* = 0.01) than without (Experiment 1, *M* = 0.69, *SE* = 0.01). The interaction of experiment and list length was not significant, *F*(2.61, 230.08) = 2.14, *p* = .105, η^2^_p_= 0.024. Critically, we found a significant two-way interaction between experiment and point-value, *F*(1.18, 103.86) = 9.51, *p* = .002, η^2^_p_= 0.10, as well as a significant three-way interaction between experiment, point-value, and list length, *F*(4.01, 353.31) = 4.31, *p* = .002, η^2^_p_= 0.047.

Pairwise comparisons suggest that the two-way interaction between experiment and point value was driven by superior recall for low-value words in Experiment 2 compared to Experiment 1, *t*(88) = 3.86, *p* = .0002, *d* = 0.81. Recall of high (*BF =* 0.28) and medium-value words (*BF* = 0.79) did not significantly differ between experiments. This indicates that when lacking foreknowledge of the list length, participants generally recalled fewer lower-value items.

Further, nested pairwise comparisons indicate that the three-way interaction between experiment, value, and list length was driven largely by the lower recall of low-value words without foreknowledge in the shorter list lengths (see Appendix Table D1 in the Supplemental Materials for all nested pairwise comparisons). Compared to Experiment 2, participants in Experiment 1 recalled a smaller proportion of low-value words in LL3 and LL6 (*p*s < .001) and in the LL9 (*p* = .04). For LL12, the typical list length used in studies of value-directed memory, there were no differences in recall between experiments for any value level (all *BF*s < 0.99). The only other significant difference between experimental conditions pertained to medium-value words in LL9 which were better recalled in Experiment 2 (*p* = .03). In sum, the results from average recall performance suggest that foreknowledge influenced how participants may have approached lower-value items in the shorter list lengths.

This conclusion is corroborated by comparing value effects on SP and OP with and without foreknowledge (see Figs. 3 and 4). For LL3, value no longer affects SP or OP when list length is known in advance. For intermediate LL6 and LL9, value effects on these two variables are somewhat attenuated when list length is known in advance, whereas for LL12 neither SPs nor OPs are affected by foreknowledge.

### Discussion

Unlike Experiment 1, Experiment 2 revealed value effects on word recall only for LL6 or more: Lists of only three words did not show any effects of value, and all three words were recalled almost perfectly. The main difference between these experiments was foreknowledge of list length, which likely allowed participants to adopt different strategies for sub-span and supra-span lists. Presumably, participants opted for alternative strategies, such as rote rehearsal, when they anticipated shorter lists which enabled them to maximize their points earned by retaining all three words. In contrast, when confronted with larger memory loads, higher value words were recalled at the expense of low-value words, an effect that was evident in average recall, across all serial positions, and by the priority output positions that characterize high- and medium-value words relative to low-value words. These patterns of results suggest that the absence of foreknowledge in Experiment 1 encouraged a more consistent value-based encoding strategy that was engaged regardless of list length. The results from Experiment 2 are also inconsistent with an automatic influence of value cues, in which case VDM effects would have been expected even for the shortest list (see also Hu et al., 2016).

A limitation of the first two experiments is ceiling level performance for the three-item lists, which was the case for the high and medium-value conditions in Experiment 1, and for all point values in Experiment 2. Ceiling-level performance restricts the range of recall accuracy for the sub-span list length. Recall accuracy was below ceiling for LL6. However, a set size of 6 is likely to be within working memory capacity for some participants, but not for others, as evident by finding that performance within this list length resembled that of LL3 in some respects (e.g., effects of foreknowledge on low-value items, and output order effects) while resembling performance of LL9 and LL12 in other respects (e.g., VDM effects regardless of foreknowledge and value-based serial position effects).

In Experiments 3 and 4, we aimed to replicate and extend the results of Experiments 1 and 2 while also addressing these limitations. To test VDM effects in working memory, we included a list length of four words instead of three, which falls within typical working memory span, but should pose greater memory demand. Further, we used a more fine-grained range of list lengths (4–11 items), to assess the continuity of VDM effects across span and supra-span lists. In addition, Experiments 3 and 4 were conducted online, and participants input their recall responses by typing the words they remembered on their keyboard. In Experiment 3, participants studied words associated with high, medium, and low point values, within varying list lengths presented in a random order and *without* advanced foreknowledge of list length (i.e., similar to Experiment 1). Experiment 4 used the same parameters as Experiment 3, except that list length was blocked and participants were informed of the list length prior to each block (i.e., similar to Experiment 2). We predicted that all list lengths, including LL4 would show VDM effects when foreknowledge was lacking, and that, as in Experiment 2, VDM effects would be selectively reduced for LL4 when list length was known in advance.

## Experiment 3

### Method

#### Participants

One hundred and two participants from the University of Michigan’s Introductory Psychology Subject Pool completed the study. An a priori power analysis was conducted using the *simr* package (v. 1.0.7; Green & MacLeod, 2016) to determine the minimum sample size required to test our hypotheses. Results indicated that an *N* of 64 was needed to have 89% power for a medium effect with a traditional ⍺ = .05 criterion of statistical significance. We oversampled to account for potential data quality concerns related to online data collection and the on-going COVID-19 global pandemic. All participants received course credit as compensation and completed the study remotely. Informed consent was obtained prior to participation. All experimental procedures were approved by the University of Michigan’s Institutional Review Board.

In contrast to the first two experiments, participants did not receive a performance-based monetary reward due to Subject Pool’s rule changes regarding monetary compensation and the online nature of the study. A total of 34 participants were excluded in the final analyses: 30 participants for indicating their data should not be included in the analyses (see *Procedure*), three participants as the result of having a less than 75% task completion rate, and one participant due to data loss. However, no participants were excluded for obtaining fewer than 250 points.

The final analyses included 68 participants^7^ (18–48 years, *M*_*age*_ = 20.07 years; see Appendix B in the Supplemental Materials for race and ethnicity).

#### Materials

To account for the novel list lengths (e.g., 4–11) included in this experiment, 98 additional words were selected, resulting in a total of 479 words used to form word lists. To reduce semantic associations found within word lists from Experiments 1 and 2, several highly associated words were replaced. All new words were selected using the same criteria and source (MRC database) as in Experiment 1. Stimuli were sorted into four practice lists (four-, five-, nine-, and 11-word lists) and 63 experimental trials, each containing four to 11 words. To minimize associations between specific words and point value categories or lists, ten different value-word lists were created for each list length and randomly assigned to participants. Each version had five list lengths (four-, five-, six-, seven-, and eight-word lists) represented with nine trials, and the remaining lists (nine-, ten-, and 11-word lists) had six trials as longer list lengths contain more words per trial. To account for this disparity, shorter list lengths were presented more frequently than longer list lengths to increase the number of observations from shorter lists.

As in Experiment 1, each word was paired with a point value ranging from 1 to 12. Point values were grouped into the three levels mentioned earlier: low (1–4 points), medium (5–8 points), and high (9–12 points). Because most list lengths used in this experiment and Experiment 4 were not divisible by 3, the point value categories were not always equally represented within a trial (i.e., for LLs 4, 5, 7, 8, 10, and 11). To address this, point value assignment was pseudo-randomized such that across the set of trials for each list length, high, medium and low point values would be equally represented.

#### Procedure

The experiment was created using PsychoPy 2.0 (Peirce et al., 2019) and uploaded to Pavlovia.org. Participants accessed the task via Pavlovia.org using a computer and internet browser. Prior to the start of the experiment, participants were instructed to minimize distractions (i.e., turn off electronic devices) and to put in their best effort. Similar to the first two experiments, participants were instructed to maximize the points they earned while also trying to recall as many words as possible from each list (Appendix Table 1 in the Supplemental Materials). Prior to the experimental trials, participants completed four practice trials consisting of four-, five-, nine-, and 11-word lists in descending order of list length. Participants were also informed that during the study, the lists would vary in length. Across experimental trials, list length varied pseudorandomly such that any particular list length did not appear more than twice in a row and word order within each trial was randomized. Similar to Experiment 1, participants had no advanced knowledge of list length and could only surmise the number of words in a given trial once the recall cue appeared.

Each trial began with a fixation star in the center of the screen (0.5 s), followed by the simultaneous presentation of a word and its associated point value for 2 s (see Fig. 1). Each word-value pair was separated by an ISI (0.5 s). To demarcate the onset of the recall period, after the last word in a list, participants saw a screen with the statement: “? ? ? Please Wait” (5 s). After the screen disappeared, responses were recorded when participants were instructed: “Please type out all of the words that you remember in lowercase letters, separated by a space. When you are done, please press ‘Enter/Return’”. The duration of the recall period was self-terminated by the participant. Participants received two brief breaks during the experiment.

Following the task, they completed a brief, open-ended exit survey (see Appendix C in the Supplemental Materials) to assess strategy use. At the end of the survey, participants were asked if their data should be included in the study in an effort to exclude participants who did not exert their best effort during the study.

### Results

**Fig. 5:**
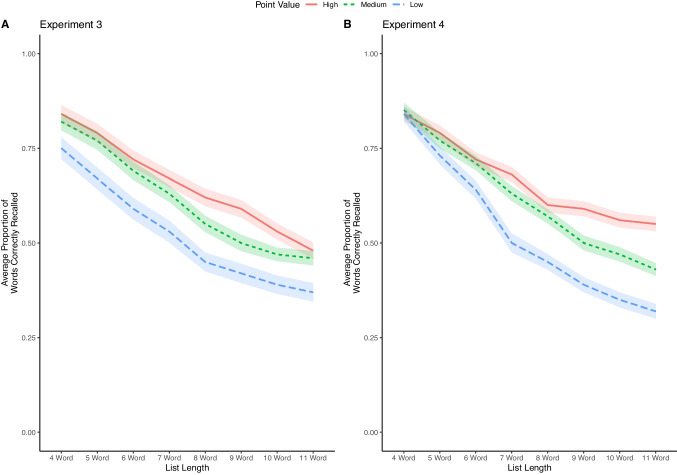
Average Proportion of Words Correctly Recalled by List Length and Point Value Category in Experiments 3 and 4. *Note.* Proportion of words recalled by list length (4–11 words) and point value: high (red, solid line), medium (green, dotted line) and low (blue, dashed line) in Experiment 3 (**A**) and Experiment 4 (**B**). In Experiment 3, participants had no foreknowledge of list length; in Experiment 4 list length was known in advance. Shaded areas around each line represent the standard error.

A 3 × 8 repeated-measures ANOVA compared the proportion of words recalled,^8^ with point category (low, medium, and high) and list length (4–11) as within-subject factors. Summary statistics are displayed in Fig. 5A. The omnibus ANOVA revealed significant main effects of list length, *F*(3.33, 222.82) = 220.02, *p* < .001, η^2^_p_= 0.77, and point value, *F*(1.18, 79.05) = 31.39, *p* < .001, η^2^_p_= 0.32. There was no significant interaction between list length and point value on the proportion of words recalled, *F*(11.98, 802.41) = 1.58, *p* = .09, η^2^_p_= 0.02.

Contrasts investigating the main effect of value revealed that participants recalled a larger proportion of high- versus medium-value words, *t*(67) = 4.54, *p* = .001, *d* = 0.54, medium- versus low-value words, *t*(67) = 5.42, *p* < .001, *d* = 0.66, and high- versus low-value words, *t*(67) = 5.86, *p* < .001, *d* = 0.71. Again, contrasts investigating the main effect of list length revealed that participants recalled a greater proportion of words as list length decreased; participants recalled a smaller proportion of words from LL11 versus LL10, *t*(67) = 4.25, *p* = .002, *d* = 0.52, LL10 versus LL9, *t*(67) = 3.99, *p* = .005, *d* = 0.49, LL9 versus LL8, *t*(67) = 4.55, *p* = .001, *d* = 0.55, LL8 versus LL7, *t*(67) = 8.09, *p* < .0001, *d* = 0.98, LL7 versus LL6, *t*(67) = 5.66, *p* < .001, *d* = 0.69, LL6 versus LL5, *t*(67) = 6.98, *p* < .0001, *d* = 0.85, and LL5 versus LL4, *t*(67) = 5.14, *p* = .0001, *d* = 0.62.

As shown in Fig. 5A, regardless of list length, a greater proportion of high-value words was recalled than low-value words, which is corroborated by a main effect of point value in the absence of an interaction with list length. Contrasts verified that the difference in recall between high- and low-value words was reliable even for the shortest list (*p*s* <* .05*;* see Table 2 for a complete set of comparisons).

**Table 2:** Comparisons of Point Value Effects on Proportion of Words Recalled for each List Length in Experiments 3 and 4 *Note.* Contrasts are presented for Experiment 3 (without foreknowledge of list length) and Experiment 4 (with foreknowledge of list length). Significant contrasts include a t-statistic (Degrees of Freedom: Experiment 3 = 67; Experiment 4 = 124), the corresponding Bonferroni corrected p-value and effect size (Cohen’s d). Strength of evidence for the null hypothesis for non-significant contrasts was assessed with Bayes Factors (*BF*). *BF* values > 1 indicate greater support for the null hypothesis; *BF* values < 1 suggest greater support for the alternative hypothesis.

	Experiment 3			Experiment 4		
	High vs. Medium	High vs. Low	Medium vs. Low	High vs. Medium	High vs. Low	Medium vs. Low
**4-word**	*BF* = 0.25	3.75 *p* = .001 *d* = 0.09	3.04 *p* = .01 *d* = 0.06	*BF* = 0.14	*BF* = 0.14	*BF* = 0.14
**5-word**	*BF =* 0.22	4.38 *p* = .001d = 0.11	4.46 *p* = .001d = 0.09	*BF =* 0.17	3.04 *p =* .01d = 0.05	*BF =* 0.24
**6-word**	*BF =* 0.26	4.71 *p* < .001d = 0.12	4.18 *p* = .0003d = 0.09	*BF =* 0.15	3.82 *p* = .001d = 0.07	4.33 *p* = .001d = 0.06
**7-word**	*BF =* 0.40	4.87 *p* < .001d = 0.13	3.67 *p* = .001d = 0.09	3.19 *p* = .005d = 0.09	6.8 *p* < .001d = 0.16	5.97 *p* < .001d = 0.12
**8-word**	3.96 *p* = .001d = 0.06	5.55 *p* < .001d = 0.15	3.91 *p* = .001d = 0.09	*BF =* 0.24	5.77 *p* < .001d = 0.14	5.63 *p* < .001d = 0.11
**9-word**	4.80 *p* < .0001d = 0.08	5.58 *p* < .001d = 0.16	3.30 *p* = .005d = 0.08	4.77 *p* < .0001d = 0.08	7.96 *p* < .001d = 0.18	6.04 *p* < .0001d = 0.1
**10-word**	3.06 *p* = .01d = 0.05	4.99 *p* < .001d = 0.13	3.96 *p* = .001d = 0.08	5.22 *p* < .0001d = 0.08	7.76 *p* < .001d = 0.19	5.92 *p* < .001d = 0.11
**11-word**	*BF =* 0.22	3.56 *p* = .002d = 0.1	4.20 *p* = .0002d = 0.08	8.06 *p* < .0001d = 0.11	8.46 *p* < .001d = 0.21	5.39 *p* < .001d = 0.1

#### Effects of value on serial position and output position

**Fig. 6:**
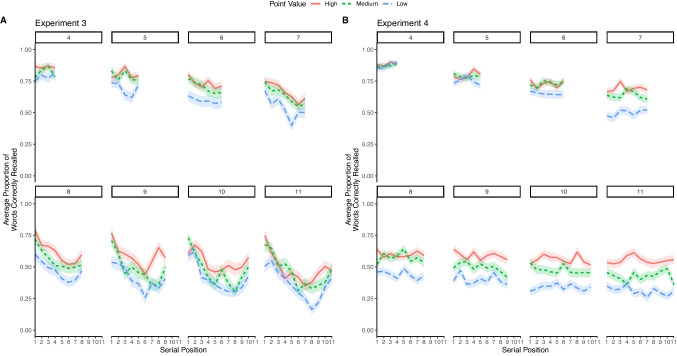
Average Proportion of Words Recalled by Serial Position, List Length, and Point Value Category in Experiments 3 and 4. *Note.* Proportion of words recalled by serial position (1–11), list length (4–11 words) and point value: high (red, solid line), medium (green, dotted line) and low (blue, dashed line) in Experiment 3 (**A**) and Experiment 4 (**B**). Shaded areas around each line represent the standard error.

**Fig. 7:**
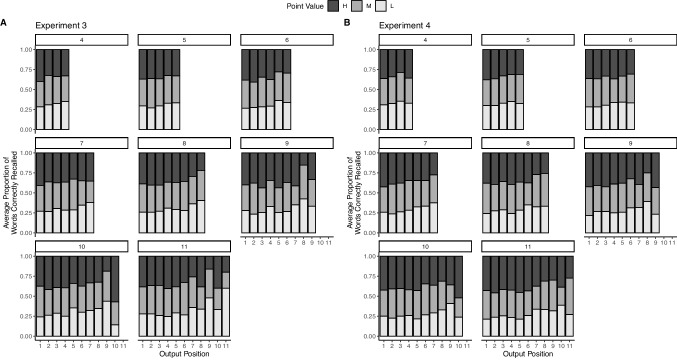
Average Proportion of Words Recalled by Output Position, List Length, and Point Value Category in Experiments 3 and 4. *Note*. Proportion of words recalled by output position (1–11), list length (4–11 words) and point value: high (dark gray), medium (gray) and low (light gray) in Experiment 3 (**A**) and Experiment 4 (**B**)

Similar to the previous experiments, the effects of value on SP and OP were calculated and plotted for each list length. As presented in Fig. 6A, value effects are evident across all list lengths and SPs, though slightly diminished in LL10 and LL11 compared to other lists. Primacy and recency effects are prominent in longer lists (LL7–LL11), whereas in shorter lists (LL4 and LL5), serial position effects are less evident. For OP plotted in Fig. 7A, it is evident that the recall of high- and medium-value words were more likely to be prioritized across all list lengths, whereas recall of low-value words increased as OP increased.

### Discussion

In the present experiment, as in Experiment 1, value effects emerged based on the proportion of words recalled for all list lengths, including the shortest list with only four words. Four items are typically considered within working memory span and constitutes the standard set size where working memory (WM) prioritization effects have been previously documented. Further, VDM effects were found for LL4 where recall was below ceiling, extending the values-based effects found with LL3 in Experiment 1 where performance approached ceiling. We speculated that the interaction of value and list length found in Experiment 1 was due to ceiling effects at the shortest list length, and the results from Experiment 3 are consistent with this interpretation in that value and list length did not interact. As in Experiment 1, better recall of high- and medium-value words relative to low-value words was generally evident across all serial positions for lists greater than five words in length. For LL4 and LL5, high- and medium-value words were better recalled than low-value words from earlier SPs, whereas the most recent SP was least sensitive to value. This pattern resembles the results from four-item memory sets in some WM prioritization studies where the most recent SP was found to be least sensitive to strategic prioritization (e.g., Hu et al., 2016). For all list lengths in the current study, recall of high- and medium-value words were prioritized for the first and second OPs, with the proportion of low-value words tending to increase with increasing OP.

Our next step was to replicate and extend the results from Experiment 2 by providing participants with advanced knowledge of list length. Thus, Experiment 4 used the same list lengths as Experiment 3 with an online sample of participants who provided typed responses. We expected that the results of Experiment 4 would be similar to those observed in Experiment 2, such that value-directed memory effects would be minimized or eliminated for the shortest list lengths when participants have foreknowledge of the size of each memory set.

## Experiment 4

### Method

#### Participants

One hundred and fifty-seven participants from the University of Michigan’s Introductory Psychology Subject Pool completed the study for course credit. Similar to Experiment 3, we oversampled to account for the possibility of poor data quality due to online data collection. Informed consent was obtained prior to participation. All experimental procedures were approved by the University of Michigan’s Institutional Review Board. Participants received course credit as compensation (see Experiment 3, *Methods* section). Thirty-two participants were excluded, of which seven participants were removed due to less than 75% task completion rate while the remaining 25 were excluded because they did not want their data to be included in the analyses (see *Procedure*). No participants were excluded for receiving fewer than 250 points. The final analyses included 125 participants (18–27 years, *M*_*age*_ = 19.13 years; 56.25% male; see Appendix B in the Supplemental Materials for race and ethnicity).

#### Materials

Materials were identical to those used in Experiment 3.

#### Procedures

The procedure was identical to Experiment 3 except for the two modifications that were also present in Experiment 2: trials were blocked by list length, and participants were informed of list length (5 s) before the onset of the block. Participants were presented with eight blocks in pseudorandom order, such that consecutive blocks were not in numerical order (e.g., participants would not see a six-word block followed by seven- and eight-word blocks). Because of the brief pauses due to the list-length information screen before the onset of each block, participants received one optional break after the first four blocks. Following the previous experiments, participants were instructed to maximize their earned points while recalling as many words as possible from each list. Table A1 in Appendix A in the Supplemental Materials reports total points earned.

### Results

A 3 × 8 repeated-measures ANOVA compared the proportion of words recalled, where point category (low, medium, and high) and list length (4–11) were within-subject factors. Summary statistics are displayed in Fig. 5B. The omnibus ANOVA revealed a main effect of list length, *F*(5.12, 634.49) = 239.64, *p* < .001, η^2^_p_= 0.66, a main effect of point value, *F*(1.24, 154.08) = 61.87, *p* < .001, η^2^_p _= 0.33, and a significant interaction between list length and point value on the proportion of words recalled, *F*(9.21, 1141.76) = 14.57, *p* < .001, η^2^_p _= 0.11.

Contrasts investigating the main effect of value on recall revealed that participants recalled a larger proportion of high- versus medium-value words, *t*(124) = 5.84, *p* < .001, *d* = 0.25, medium- versus low-value words, *t*(124) = 7.96, *p* < .001, *d* = 0.47, and high- versus low-value words, *t*(124) = 8.28, *p* < .001, *d* = 0.71. Contrasts investigating the main effect of list length revealed that proportion recalled increased as list length decreased. Participants recalled a smaller proportion of words from LL10 versus LL9, *t*(124) = 3.29, *p* = .04, *d* = 0.22, LL9 versus LL8, *t*(124) = 3.77, *p* = .007, *d* = 0.26, LL8 versus LL7, *t*(124) = 5.89, *p* < .001, *d* = 0.35, LL7 versus LL6, *t*(124) = 6.86, *p* < .001, *d* = 0.44, LL6 versus LL5, *t*(124) = 5.46, *p* < .001, *d* = 0.34, and LL5 versus LL4, *t*(124) = 5.26, *p* < .001, *d* = 0.39. There was no significant difference in recall rates between LL11 (*M = *0.43*, SE =* 0.01) and LL10 (*M =* 0.46, *SE =* 0.01; *BF* = 0.29, *p* = .23).

These main effects were qualified by an interaction of point value and list length. As is evident from the means in Fig. 5B and the paired comparisons reported in Table 2, more high-than low-value words were recalled for all list lengths except for the shortest list length of four items, where the proportion of recall did not differ among any of the point values.

#### Effects of value on serial position and output position

To further examine the effect of value, we computed the proportion of words recalled as a function of SP and OP. As presented in Fig. 6B, value effects are no longer evident across any SPs for LL4 corroborating the results from proportion recalled. For LL5, value effects are evident for primacy and recency positions, but not for middle SPs. For all other list lengths, even though primacy and recency effects are muted, the proportion of high- and medium-value words recalled exceeds that of low-value words across SPs. Effects of value on OP can be seen in Figure 7B. For LL5 through LL11, the recall of high- and medium-value words is prominent in the first few OP. However, for LL4, the effect of value is attenuated in comparison to other list lengths, and in comparison with LL4 without foreknowledge. For LL6 and greater, the recall of low-value words is least likely in the first few OPs but increases with increasing OP.

### Comparison of Experiments 3 and 4

Again we examined the effect of foreknowledge on recall by statistically comparing Experiments 3 and 4. A 2 × 3 × 8 mixed-design ANOVA compared the proportion of words recalled, where experiment (3 vs. 4) was the between-subject factor and value (high, medium, and low) and list length (4–11) were within-subject factors. As in the main analyses for Experiments 3 and 4, the omnibus test indicated significant main effects of point value, *F*(1.22, 233.90) = 85.16, *p* < .001, η^2^_p_= 0.306, and list length, *F*(4.84, 925.03) = 370.08, *p* < .001, η^2^_p_= 0.66, and a significant two-way interaction between point value and list length, *F*(10.68, 2040.68) = 8.32, *p* < .001, η^2^_p_= 0.042. Neither the main effect of experiment, *F*(1, 191) = 0.09, *p* = .77, η^2^_p_< 0.001, nor either of the two-way interactions were significant: experiment and point value *F*(1.22, 233.90) = 0.05, *p* = .86, η^2^_p_< 0.001, and experiment and list length, *F*(4.84, 925.03) = 1.65, *p* = .15, η^2^_p_= 0.009.

However, the three-way interaction between experiment, point value, and list length was significant, *F*(10.68, 2040.68) = 4.82, *p* < .001, η^2^_p_= 0.03. Nested pairwise comparisons suggest that this three-way interaction is due to foreknowledge influencing the recall of low-value items in the shortest list length, and high-value items in the longest list length. Participants in Experiment 4 (i.e., with foreknowledge) recalled larger proportions of low-value words in LL4 (*p* = .01) and more high-value words in the LL11 (*p* = .02) compared to participants in Experiment 3 (i.e., without foreknowledge). Recall did not significantly differ between experiments for any other combination of point-value and list length condition (*BF*s < 0.53; *p*s > 0.05). The full set of nested pairwise comparisons can be found in Appendix Table D2 in the Supplemental Materials. In sum, these results from average recall performance suggest that foreknowledge may have promoted participants to engage different strategies specifically in response to the shortest list compared to lists of five words or more.

This interpretation is further supported by comparing the effect of value on SP and OP between experiments. With foreknowledge, value no longer affects SP nor prominently affects OP within the shortest list (LL4). However, as list lengths increase, the effect of value is largely present in both SP and OP whether or not foreknowledge of list length was provided.

### Discussion

Experiment 4 replicated key results from Experiment 2: When participants could anticipate list length, there was no evidence of VDM effects in average proportion recalled for lists within typical working memory capacity (i.e., four-word lists). Moreover, for lists of five items or more, VDM effects persisted even with foreknowledge. In particular, as with the previous pair of studies, comparison of Experiments 3 and 4 indicates that when studying LL4 without foreknowledge of list length, participants recall more high- and medium-value words, while ignoring or forgetting low-value words, as further reflected in the serial position effects. However, uniquely for LL4, this tendency disappears when participants can anticipate the forthcoming memory load, in which case assigned point value has no influence on average recall of high, medium, and low-value words, which is equivalent across value levels. Indeed, with foreknowledge, words from LL4 at each value level are recalled as well as high-value words when foreknowledge is lacking. In Experiment 2, recall of all words from the LL3 was also equally high, and approximated recall performance for high- and medium-value words in Experiment 1, where foreknowledge was lacking. However, with performance well below ceiling, comparing average recall and serial position effects as a function of value for Experiments 3 and 4 makes evident that rather than improved recall of high-value words, VDM effects for short lists were primarily due to poorer recall of low-value words.

Overall, results from Experiments 3 and 4 replicate the results from Experiments 1 and 2, respectively. Value-directed effects are evident for sub-span and near-span memory loads when list length cannot be anticipated. However, with foreknowledge of list length, VDM effects are no longer evident for lists lengths within or near working memory capacity (i.e., set size 4).

## General discussion

The present study investigated the effects of value cues associated with to-be-remembered words presented in a range of list lengths including those within or near canonical limits of working memory. In four experiments word-value pairs were presented sequentially; in Experiments 1 and 2, participants studied lists of 3–12 words and in Experiments 3 and 4, participants studied lists of 4–11 words. A key design feature of Experiments 1 and 3 was that list length was randomized and unknown to the participant at the start of each trial. In contrast, in Experiments 2 and 4, list length was blocked and participants had foreknowledge of the number of word-value pairs in each list. Without foreknowledge, VDM effects were evident in proportion of words recalled for all list lengths: Participants were more likely to recall words associated with high than low point values for all list lengths including those with three or four items that fall within the typical span of working memory. This pattern was observed when participants were tested in person or online, whether or not points were associated with monetary rewards, and regardless of whether recall responses were spoken aloud, or typed. In contrast, when list length was known in advance, VDM effects were only evident for lists of five items or *greater*, suggesting that under these conditions, participants opted not to use value cues for remembering words in the shortest lists (i.e., three to four items).

The unique effects of foreknowledge on VDM effects for lists of three or four items was further evident in serial position effects and output order in recall. Without foreknowledge (Experiments 1 and 3) low-value words were more poorly recalled than high- and/or medium-value words from all SPs except the most recent, whereas with foreknowledge no value effects were evident for any SPs for three- and four-word lists. Examination of the response output sequences for three- and four-word lists similarly indicated that low-value words were least likely to be among the earliest words recalled when foreknowledge was lacking whereas with foreknowledge, value had no effect on output order for these shorter lists. For lists of five or more words, the values effects on SP and OP were relatively unchanged by the availability of foreknowledge: in general low-value words were least likely to be recalled from all SPs, and were least likely to be among the earliest words recalled, with a general trend toward increasing likelihood of their recall with increasing OP.

The present results from Experiments 1 and 3 extend the evidence for prioritization effects in working memory by demonstrating VDM effects for free recall of words presented sequentially in lists of three or four items with dynamically changing value cues. Furthermore, these findings expand the lower range of list lengths for which VDM effects are typically found (i.e., ten or more items) to include lists ranging from three to nine words (Castel et al., 2002; Castel et al., 2007, Murphy et al., 2025). Indeed, we posit that when lacking foreknowledge of list length, participants adopted a more uniform encoding strategy for shorter and longer lists directed by value cues. That is, because initial list items were potential members of a supra-span list, participants were more likely to use value-directed strategies engaged for long-term memory within the parameters of a working memory task. Such strategies could include preferential rehearsal of higher value items, deeper associative processing or both (e.g., Knowlton & Castel, 2021; Stefanidi, et al., 2018). The continuity of VDM effects across short and long list lengths is further evident in SP curves that are graded by value, and in the patterns of output, whereby high- and medium-value words are most likely to be recalled as the first responses generated and then steadily diminish over output sequence, whereas low-value words are more likely to be recalled later in the output sequence. Accordingly, the results from Experiments 1 and 3 suggest the possibility that dynamic control processes that yield value-directed effects in lengthy lists may be brought to bear on lists that fall within working memory capacity, consistent with embedded process models of memory (Cowan, 1999).

In contrast, the results from Experiments 2 and 4 indicate that when list length was known in advance, different strategies were engaged to remember words in sub-span versus supra-span lists. High-value items were better recalled than low-value items for all list lengths, except for lists of three or four words where VDM effects were no longer evident and recall accuracy was roughly equivalent regardless of word value. Likewise, plotting SP and OP indicates virtually no effects of value for lists of three or four words, whereas all other list lengths show largely similar value effects on SP and OP with and without foreknowledge. As noted, the absence of value effects for three- and four-word lists in the foreknowledge conditions is due primarily to improved memory for low-value words. That is, the presence of value effects for these list lengths when foreknowledge is lacking comes at a memory cost for low-value words that is evident across all measures. In the following sections, we consider the potential strategic bases for VDM effects in shorter and longer lists, how they might differ, and how the present results relate to prioritization effects in working memory. Limitations of the present work, and directions for future research are also considered.

### The effect of foreknowledge on recall from lists of three and four words

Without foreknowledge of list length, pronounced VDM effects were evident for lists within working memory span, but when list length could be anticipated, VDM effects disappeared. Why might this be? Coupled with the lack of VDM effects, the foreknowledge conditions had better recall overall, with a specific boost in memory for low-value words in shorter lists. Could this reflect a tendency for participants to invest greater effort in the memory task with foreknowledge than without? That is, when lacking foreknowledge might greater ambiguity or uncertainty about list length lead to reduced effort, resulting in greater memory *cost* for low-value words, and an associated increase in VDM effects for shorter lists? If so, we might also expect larger VDM effects in Experiment 3 than Experiment 1 because the former experiment had more ambiguity due to greater variation in list lengths used in that study. Comparing mean recall of high minus low value words (i.e., the VDM effect) for LL3/Experiment 1 (*M* = 0.11) to LL4/Experiment 3 (*M* = 0.09) indicates minimal differences in the magnitude of the VDM effects, although these effects are numerically smaller under conditions of greater ambiguity (see Figs. 2 and 5). Note, however, the learning and memory literature more broadly suggests that ambiguity and associated increases in task difficulty can prompt an *increase* in effort and motivation (e.g., Bjork & Bjork, 2011; Gershman, 2018). Taken together, we surmise that poorer memory for low-value words in shorter lists without prior knowledge of list length is unlikely to result from decreased effort in ambiguous conditions, although this possibility merits further investigation.

We posit instead that value-based encoding strategies are themselves engaged strategically depending on foreknowledge of working memory load, underscoring the influence of metamemory (Atkinson et al., 2019; Knowlton & Castel, 2022) on VDM effects especially for shorter lists. Without foreknowledge there is continuity in the engagement of value-directed strategies regardless of list length as evidenced by proportion recalled, and serial position and output sequence patterns. In contrast, when list length was known in advance, the selective absence of VDM effects suggests that participants opted not to use value cues for remembering words in lists they judged to be within their working memory span, instead engaging rote rehearsal or possibly chunking of list items (e.g., Thalmann et al., 2019), resulting in an overall improvement in memory for words in lists of three or four items.

### A cost of value-based encoding for short lists?

When participants could anticipate a shorter list, all value levels were recalled equally well and as accurately as high-value words without foreknowledge of list length. However, without foreknowledge recall was poorer for low-value words, with no further benefit to higher value words. The absence or diminution of VDM effects for three- and four-word lists with foreknowledge (i.e., Experiments 2 and 4) was due primarily to *better* recall of low-value words compared to when foreknowledge was lacking, suggesting that use of a value-directed strategy incurred a cost for lower-value cues.

This possibility is noteworthy for several reasons. First, as discussed previously, VDM effects with longer lists are thought to result from deeper processing of higher value items, shallower processing of lower value items, or a combination of these two strategies. Previous research using eye tracking and pupillary response has shown that participants fixate on high- and low-value words for similar durations, but their eyes dilate more when studying high-value words, suggesting that value-directed effects are due to enhanced encoding of high-value words (Ariel & Castel, 2014). The present results suggest this may not be the case for shorter lists because VDM effects emerged due to poorer recall for low-value items, rather than better memory for high-value items that is evident with longer lists.

Furthermore, finding that VDM effects for three- and four-word lists were associated with a memory cost for low-value words, without a memory benefit for higher value words contrasts with a dominant finding in the working memory prioritization literature where value typically redistributes attentional resources (e.g., Allen et al., 2024; Atkinson et al., 2021; Hu et al., 2014). That is, prioritization studies using verbal materials often show a trade-off in resource allocation coupled with no net change in overall memory performance: high priority items are associated with better memory, relative to items of lower or neutral priority, without an overall effect on performance relative to “equal value” control conditions without prioritization (e.g., Sandry et al., 2014; Sandry et al., 2020). In contrast, our results suggest that when using value to retain lists of three to four words participants may have strategically reduced the memory resources allocated to low-value items which suffered a recall drop of more than 10% in Experiments 1 and 3 when foreknowledge of memory load was absent. A related possibility is that rather than reducing resources allocated to low-value words, participants reserved or invested greater resources in memory for high-value words. However, the cost to low-value words was incurred without a corresponding boost to high-value words: Recall accuracy for high-value words was unaffected by foreknowledge within shorter lists. This pattern suggests that lower value words are more likely to be ignored or forgotten from working memory when foreknowledge of list length was lacking.

This discrepancy between the present VDM effects with shorter lists and some results from studies of prioritization in WM could stem from task differences, associated differences in strategic demands (e.g., Atkinson et al., 2021), or a combination of factors. One possibility to consider is overall differences in task difficulty. Some prioritization paradigms that find a resource redistribution pattern using verbal or non-verbal materials (Atkinson et al., 2018; Hu et al., 2016; Sandry et al., 2014) may place greater demands on feature binding and have lower overall performance (e.g., in the probe-recognition tasks) compared to the present work. However, within the current data set, we can assess whether use of value cues for shorter lists, and corresponding VDM effects, are more pronounced for harder conditions by again comparing effects for three- versus four-item lists, in Experiments 1 and 3, respectively. Whereas overall recall accuracy was 94% and 80%, respectively, indicating substantial differences in difficulty, VDM effects are comparable across the two studies: 0.11 for LL3 in Experiment 1 and 0.09 for LL4 in Experiment 3. This pattern suggests that differences between the current VDM effects and prioritization effects in working memory are not simply due to differences in difficulty.

Assigning value in the current tasks likely entails strategic and cognitive/executive control demands that differ from those more commonly used in WM prioritization studies (see also Allen et al., 2024; Atkinson et al., 2021; Hu et al., 2016). Specifically, the present task requires dynamic processing and binding of multiple value-word pairings per trial unlike WM prioritization trials which more typically involve a single high-priority item. The cognitive control required to compare successive values and update the memory set, could compromise item encoding or storage capacity, resulting in a memory cost for low-value words without a corresponding boost for higher value words (cf., Hu et al., 2016).

### The effect of foreknowledge on recall from longer, supra-span lists

Comparison of Experiments 1 and 2 indicates that whether or not participants had foreknowledge of list length, VDM effects were significant and unchanged for LL12. For LL6 and LL9, VDM effects were evident regardless of foreknowledge; however, *lower* value words were better recalled when list length was known in advance. A different pattern is evident when comparing supra-span lists in Experiments 3 and 4. In this case VDM was unaffected by foreknowledge (for LL5–10), with the exception of LL11 where recall of high-value words was better when list length was known in advance. Thus, across the four studies, for supra-span lists, foreknowledge has differing effects on memory for high- and low-value words. One reason for this inconsistency may be that the conditions in Experiments 3 and 4 were overall more challenging than in the first two studies. Not only was there greater variation in list length in the latter two studies, but recall required typing responses rather than merely saying them aloud. These procedural differences are associated with a drop in recall performance by at least ten percentage points as evident by comparing recall accuracy for LL6 and LL9, which were common across all four experiments.

Overall, the results demonstrate that VDM effects are robust for longer lists, with some strategic variation in treatment of high- and low-value words based on foreknowledge of memory load, and recall conditions that affect task difficulty. The present results suggest these strategies can vary from increased emphasis on high-value words, presumably due to more elaborative and associative processing, to relative inattention to and forgetting of lower value words.

## Limitations and future directions

Several limitations of the present research warrant consideration. These experiments relied on well-established span estimates to designate list lengths falling within or exceeding verbal working memory capacity. Future research could estimate working memory span individually and examine value-directed memory effects for lists within or exceeding each participant’s working memory span. Additionally, more work is needed to understand variations in strategy use due to the presence or absence of foreknowledge and list length. That is, in the absence of foreknowledge, does processing depth differ for high- and low-value words regardless of list length? The surveys used in the current study were administered at the end of each experiment, showed no significant variation due to foreknowledge, and lacked nuanced information about potential variations in strategy use due to list length (see Appendix C in the Supplemental Materials). However, if probes about strategy use had been more frequent or embedded in the task, participants' approach to the task would likely have been altered.

Furthermore, some caution is warranted when interpreting the cross-experimental comparisons. Inferences about the effects of foreknowledge, ambiguity/effort, and task difficulty were drawn from between-experiment comparisons. However, these experiments were conducted consecutively without random assignment of participants to the different experimental conditions. As a result, the possibility of group-level differences contributing to the observed effects cannot be ruled out. Future work can address this limitation by replicating these experiments with random assignment or using a within-subjects approach.

Better subsequent long-term memory for high-value or prioritized words could also provide an index of processing depth (see, e.g., Sandry et al., 2020). To test the possibility of enduring effects of value-based encoding across list lengths, Experiments 1 and 2 of the current study included a surprise long-term recall test administered after all VDM trials were completed; however, recall was so poor and variable that these data were uninformative and could not be analyzed as a function of list length (Jantz, 2018). Indirect measures, including measurement of pupil diameter and functional brain imaging, would provide methods of monitoring strategy that are less likely to alter the strategies themselves and reveal potential differences due to value, list length, foreknowledge, and recall mode.

Finally, the current finding that value-based memory for shorter lists is associated with a performance cost for low-value words without a corresponding benefit to high-value words, suggests that, unlike other working memory prioritization paradigms (e.g., Allen et al., 2024), some cost to memory capacity is incurred by the processing demands of the present VDM task. Future research that evaluates such task differences and associated strategy effects could address questions about different mechanisms of prioritization, their resource demands, and potential consequences for memory performance over the short and longer term.

## Conclusion

The current research provides evidence that value cues can be used effectively to influence recall of items from lists within or near working memory span, suggesting the possibility that memory control processes known to benefit long-term memory of “valuable” words can operate within the canonical parameters of working memory. However, the results suggest that these control processes are engaged strategically and largely abandoned for shorter lists when people have advanced knowledge of the forthcoming memory load, reflecting the availability of alternative, more efficient strategies for list lengths within working memory span. That is, for set sizes of three or four, assessments of memory load and metamemory judgements of one’s ability to retain that load are likely key determinants of how participants engage with value cues for shorter lists. Moreover, VDM effects for shorter lists emerge at the expense of low-value items, whereas VDM effects for longer lists also show evidence of enhanced processing of high-value words. Thus, while the present results demonstrate VDM effects regardless of list length under some conditions consistent with unitary models of memory, discontinuities in these effects indicate the availability of alternative strategies for remembering lists at or near working memory span.

## Supplementary Information

Below is the link to the electronic supplementary material.

Supplementary file1 (DOCX 95 KB)

## Data Availability

Data for all experiments can be found on the Open Science Framework (OSF): https://osf.io/tnfse/. Additional task materials are available upon request.

## Footnotes

1 The amount of information that can be actively processed in working memory, for example, its capacity, is an area of ongoing debate (Cowan, 2001). The term “span” is commonly used to describe working memory capacity for verbal (e.g., digits, letters, or words) information (Barrouillet et al., 2021; Conway et al., 2005). In this paper, we use the terms capacity and span interchangeably.

2 Data for Experiments 1 and 2 were collected as a part of Jantz (2018).

3 In Experiments 1 and 2, participants were asked whether they identified as male or female with no nonbinary option. Participants were not asked to disclose whether they were cisgender or transgender.

4 Phonetically similar responses and repetitions were not included in analyses, and therefore are not further discussed.

5 We opted not to use selectivity indices due to near ceiling performance in some list length conditions, varying list lengths, and associated differences in point values, all of which affect this index and the validity of comparisons across conditions (see, e.g., Murphy, 2023).

6 Analyses of SP and OP were post hoc and included during the editorial process.

7 Due to experimenter error, information about gender was not collected.

8 Similar to the previous experiments, phonetically similar or misspelled responses were not included in the analyses.

## Code availability

All analyses were conducted on R Studio and code for primary analyses can be found on the OSF: https://osf.io/tnfse/.
