# Supplementary material for: Strategy effects and value directed recall of sub- and supra-span word lists
Source: Mem Cognit. 2026 May 19;54(5):1609–31. doi: 10.3758/s13421-025-01828-4 (PMC13407582; doi:10.3758/s13421-025-01828-4)
Supplement: Supplementary file 1 — Supplementary file1 (DOCX 95 KB) [file 13421_2025_1828_MOESM1_ESM.docx]

**Appendix A**

**Comparison of Points Earned Across Experiments**

In these VDM tasks, participants were instructed to maximize the number of point-values earned while trying to recall as many words as possible. The point values of each word correctly recalled were summed across trials to tally the points scored for each participants. In Experiments 1 and 2, participants were eligible to receive a monetary reward if their total score was at least 250 points. We opted to pay participants based on a fixed and relatively low threshold of 250 points simply to call attention to the point values and encourage engagement with the points. Additionally, setting a relatively attainable threshold ensured that most participants could receive the additional five USD bonus payment to mitigate disparities in compensation, which was the preferred approach for use of our subject pool. In Experiments 3 and 4, participants were not provided with this monetary incentive. Participants in all experiments were excluded from the study if their score was less than 250 points, indicating insufficient effort exerted during the task.

The total number of possible points earned varied depending on experiment (see Table A1 below) due to variations in list lengths and differences in point value assignments. In Experiments 1 and 2, each word had an associated point-value that remained constant across participants. In Experiments 3 and 4, different point values were associated with different words, such that the word-value pair varied across participants. To account for this variability in total possible points, we calculated the proportion of points earned by dividing the number of points earned by the total possible points. Unsurprisingly, across experiments, the proportion of points earned was strongly correlated with overall recall accuracy, *r*(280) = 0.93, p < .001. This close relationship between total points earned and overall recall accuracy motivates using total points earned to exclude the few low-earning participants from the analysis.

To compare whether the proportion of points earned differed across experiments, we ran a between subjects analysis of variance with type III sum of squares using the *aov_ez* function (version 1.3-0; Singmann et al., 2023) in R. We found a significant main effect of experiment, *F*(3, 278) = 8.8447, *p* < .001. Post-hoc pairwise comparisons with Bonferroni-corrections demonstrated that the proportion of points earned was significantly higher in the first two

experiments compared to the second two experiments. Participants in Experiment 1 earned a larger proportion of points compared to Experiment 3, *t*(100.60) = 5.51, *p* < .001, and Experiment 4, *t*(168.35) = 3.70, *p* = .002. Similarly, participants in Experiment 2 earned a larger proportion of points compared to Experiment 3, *t*(108.74) = 5.13, *p* < .001, and Experiment 4, *t*(130.12) = 3.48, *p* = .004. There was no significant difference in the proportion of points earned in Experiment 1 compared to 2, *t*(74.17) = -0.37, *p* = .712. Finally, there was a marginally significant difference in the proportion of points earned in Experiment 3 compared to 4, *t*(157.04) = -1.90, *p* = .06, with participants in Experiment 4 earning a slightly higher proportion of points compared to Experiment 3. Taken together, the difference in points earned between the first and second set of experiments supports the inference that the online experiments were more difficult than the in-person experiments. However, we cannot rule out that the difference in monetary incentives also played a role, due to the limited scope of the survey questionnaires (see Appendix C).

**Table A1**

*Summary Statistics on Points Earned and Possible Points Earned Across Experiments*

| **Experiment** |  | **Earned points** | |  |  | **Possible points** | |  |
| --- | --- | --- | --- | --- | --- | --- | --- | --- |
|  | *Mean* | *SD* | *Min* | *Max* | *Mean* | *SD* | *Min* | *Max* |
| 1 | 1585.67 | 146.39 | 1118 | 1853 | 2340 | 0 | 2340 | 2340 |
| 2 | 1600.05 | 211.44 | 1145 | 2042 | 2340 | 0 | 2340 | 2340 |
| 3 | 1781.63 | 424.97 | 538 | 2710 | 3107.93 | 15.32 | 3065 | 3135 |
| 4 | 1798.60 | 469.33 | 543 | 2896 | 2925.37 | 15.78 | 2888 | 2968 |

*Note*. SD = standard deviation

**Figure A1**

*Proportion of Points Earned Across Experiments*

*
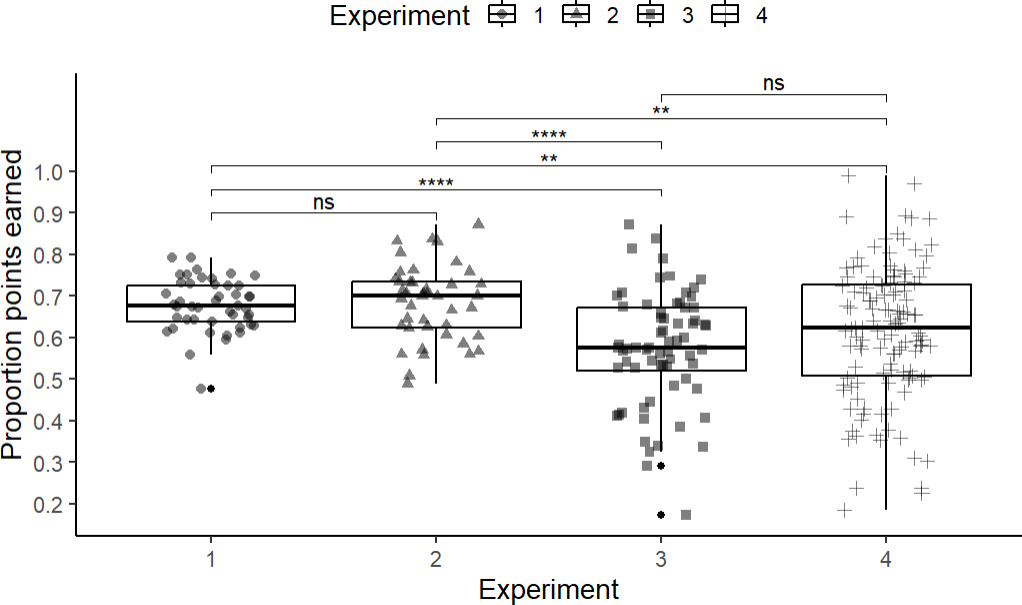
*

*Note*. ns = no significant difference; **= *p* < .01, ****p* < .001

**Appendix B**

**Participant Demographics**

**Table B1**

*Participant Demographics for Experiments 1, 2, 3, and 4*

|  | **Experiment 1** |  | **Experiment 2** |  | **Experiment 3** |  | **Experiment 4** |
| --- | --- | --- | --- | --- | --- | --- | --- |
| **Number of participants** | 46 |  | 44 |  | 68 |  | 125 |
| **Age (years)** | 18.93 |  | 18.86 |  | 20.07 |  | 19.14 |
| **Education (years)** | 13.04 |  | 12.82 |  | 13.33 |  | 12.75 |
| **Gender** | 30.4% F, 69.6% M |  | 54.5% F , 45.5% M |  | 8.8% F, 17.6% M,  73.5% NR |  | 43.2% F, 54.4% M,  1.6% NB, 0.8% NR |
| **Race** | 4.3% A, 2.2%  MENA, 52.2%  NR, 41.3% W |  | 11.4% A, 6.8% B,  2.3% NR, 79.5% W |  | 16.2% A, 8.8% B,  2.9% NR, 1.5%  NWH, 70.6% W |  | 27.2% A, 6.4% B 3.2%  MENA, 1.6% MR,  1.6% NR, 1.6% NWH,  58.4% W |

*Note.* Gender includes F = female, M = male, NR = not reported, NB = nonbinary. Races include A = Asian or Asian American, B = Black or African American; MENA = Middle Eastern or North African, MR = Multiracial, NR = Not reported, NWH = Non-White Hispanic, W = White. Experimenter error, data loss, or lack of participant disclosure led to not reported gender or race.

**Appendix C**

**Exit Surveys and Responses Across Experiments**

As reported in the Methods section of the main text (see Experiments 1, 2, 3 and 4), following the computerized task, participants completed a computerized exit survey composed mainly of open-ended questions. The exit surveys were aimed to gather information about strategy use and how these strategies varied by experiment. Two graders independently coded and categorized the responses into thematic groups of strategies. Afterward, the graders compared their categorization of participant responses and discussed discrepancies to reach a consensus. If participants used two strategies simultaneously, they were categorized into both strategy groups. Because the exit surveys' questions varied across experiments, we specifically compared responses to the question, ‘‘What effect, if any, do you feel the point value had on your overall memory performance?’’. After assessing the strategies used in the experiments, responses were grouped into three main categories: participants who reportedly did not use points, participants who focused on high points, and participants who ignored low points.

To examine the effect of foreknowledge (i.e., Experiments 1 vs 2 and Experiments 3 vs 4) and task difficulty (i.e., Experiments 1 vs 3 and Experiments 2 vs 4) on the proportion of self-reported strategies, we ran a series of two-sample chi-square tests using the *stats* function in R (version 4.3; R Core Team, 2013). Bonferroni corrections were applied on the resulting *p*-values. Table C1 depicts the percent and standard deviation of reported strategies by experiment while Table C2 reports the results of the chi-square tests. All comparisons were non-significant after correcting for multiple comparisons (see Table C2).

**Table C1**

*Proportion and Standard Deviation of Self-Reported Strategies*

|  |  | **Strategy** |  |
| --- | --- | --- | --- |
| **Experiment** | Ignored All Points | Focused on High Points | Ignored Low Points |
| 1 | 33.0% (7.0%) | 50.0% (7.4%) | 17.4% (5.6%) |
| 2 | 40.4% (7.6%) | 36.0% (7.4%) | 24.0% (6.6%) |
| 3 | 49.2% (6.0%) | 42.2% (5.9%) | 8.4% (3.3%) |
| 4 | 53.0% (4.5%) | 38.0% (4.4%) | 9.1% (2.6%) |

*Note.* Standard deviation in parentheses.

**Table C2**

*Detailed Results Between Experiment 1 - 4 on Proportion of Reported Strategies*

| **Strategy** | **Experiment Comparisons** | **Chi-Square** | **Bonferroni Corrected p-value** |
| --- | --- | --- | --- |
| Ignored All Points | 1 vs 2 | 0.30 | 1.00 |
|  | 3 vs 4 | 0.11 | 1.00 |
|  | 1 vs 3 | 2.53 | .22 |
|  | 2 vs 4 | 1.46 | .45 |
| Focused on High Points | 1 vs 2 | 1.29 | .51 |
|  | 3 vs 4 | 0.18 | 1.00 |
|  | 1 vs 3 | 0.40 | 1.00 |
|  | 2 vs 4 | 0.006 | 1.00 |

| Ignored Low Points | 1 vs 2 | 0.23 | 1.00 |
| --- | --- | --- | --- |
|  | 3 vs 4 | < 0.001 | 1.00 |
|  | 1 vs 3 | 1.35 | .49 |
|  | 2 vs 4 | 4.78 | .06 |

**Experiment 1 and 2: Survey Instrument**

Thank you for your participation. As part of our research, we are interested in your input and impressions of the experiment you have just completed. Please answer the following questions to the best of your ability.

1. Did you use any strategies, short cuts, or “tricks” to help you in the first or second memory task? If so, explain.
2. If you used strategies during the first memory task, what effect, if any, did the point value have on those strategies?
3. What effect, if any, do you feel the point value had on your overall memory performance?
4. Did you think that certain parts of the first memory task were more difficult than others? If so, explain.
5. After the first memory task, were you surprised that we asked you to remember which words you had seen before?
6. Did you try harder to remember words on trials with high point values?
7. Did you feel that point values ever interfered with your ability to remember the words? (Please explain you answer)
8. Were there any additional factors that may have affected your performance on any of the tasks, such as your own fatigue, noise in the lab, or other distractors?
9. What do you think this experiment was testing?

**Experiment 3 and 4: Survey Instrument**

Thank you for your participation. As part of our research, we are interested in your input and impressions of the experiment you have just completed. Please answer the following questions to the best of your ability.

1. Did you use any strategies, short cuts, or “tricks” to help you in the first memory task? If so, explain.
2. If you used strategies during the first memory task, what effect, if any, did the point value have on those strategies?
3. What effect, if any, do you feel the point value had on your overall memory performance?
4. Did you think that certain parts of the first memory task were more difficult than others? If so, explain.
5. **{This question was only asked in Experiment 4}** What effect, if any, did knowing how long the lists were had on the strategies that you used to remember the words?
6. Did you try harder to remember words on trials with higher point values?
7. Did you feel that the point values ever interfered with your ability to remember the words? (Please explain your answer)
8. Were there any additional factors that may have affected your performance on any of the tasks, such as your own fatigue, noise in the lab, or other distractors?
9. What do you think this experiment was testing?

**Appendix D**

**The Effect of Foreknowledge on Value and List Length Across Experiments**

As reported in the main text (see Experiment 2 Results and Experiment 4 Results), we compared whether foreknowledge influenced the use of value cues across list lengths. More specifically, we examined whether value-directed memory effects present at the shorter list lengths in experiments lacking foreknowledge (e.g., Experiments 1 and 3) were due to better recall of high-value items, poorer recall of low-value items or a combination of the two.

We compared Experiments 1 and 2 using a 2 (Experiment) x 3 (Point value) x 4 (List length) analysis of variance. Similarly, we compared Experiments 3 and 4 using a 2 (Experiment) x 3 (Point value) x 8 (List length) analysis of variance. We found significant three-way interactions between experiment, point value, and list length in both omnibus analyses. To compare the difference in recall within each pair of experiments for each list length and point value combination, we used the *emmeans* function (version 1.10.0; Length, 2024) in R to conduct post-hoc nested pairwise comparisons. Bonferroni corrections on the resulting *p*-values were applied to correct for multiple comparisons. Table D1 reports results comparing Experiments 1 and 2. Table D2 reports results comparing Experiments 3 and 4.

**Table D1**

*Pairwise Comparisons Between Experiments 1 and 2 Within Each Combination of List Length and Point Value on Proportion of Words Recalled*

| **List length** | **Point value** | **Difference**  **(Exp1-Exp2)** | **T-statistic** | **DF** | **Corrected**  ***p-*value** | **Bayes Factor** |
| --- | --- | --- | --- | --- | --- | --- |
| **3** | **Low (1-4)** | **-0.117** | **-4.94** | **88** | **< .0001** | **4233.39** |
|  | Med (5-8) | -0.010 | -0.98 | 88 | .328 | 0.34 |
|  | High (9-12) | -0.012 | -1.63 | 88 | .1071 | 0.7 |
| **6** | **Low (1-4)** | **-0.202** | **-4.78** | **88** | **< .0001** | **2365.45** |
|  | Med (5-8) | -0.032 | -1.32 | 88 | .191 | 0.47 |
|  | High (9-12) | 0.032 | 1.45 | 88 | .1502 | 0.56 |
| **9** | **Low (1-4)** | **-0.094** | **-2.14** | **88** | **.0355** | **1.6** |
|  | **Med (5-8)** | **-0.057** | **-2.22** | **88** | **.0288** | **1.88** |
|  | High (9-12) | 0.032 | 1.18 | 88 | .241 | 0.41 |
| **12** | Low (1-4) | -0.069 | -1.85 | 88 | .0673 | 0.99 |
|  | Med (5-8) | -0.009 | -0.39 | 88 | .7014 | 0.24 |
|  | High (9-12) | 0.003 | 0.09 | 88 | .9277 | 0.22 |

*Note.* DF = degrees of freedom. P-values corrected using the Bonferroni method. Values in bold indicate significant differences in recall between experiments. Negative difference values reflect higher recall for Experiment 2 compared to 1; positive difference values reflect higher recall for Experiment 1 compared to 2.

**Table D2**

*Pairwise Comparisons Between Experiments 3 and 4 Within Each Combination of List Length and Point Value on Proportion of Words Recalled*

| **List length** | **Point value** | **Difference**  **(Exp3-Exp4)** | **T-statistic** | **DF** | **Corrected**  ***p-*value** | **Bayes Factor** |
| --- | --- | --- | --- | --- | --- | --- |
| 4 | **Low (1-4)** | **-0.092** | **-2.70** | **191** | **.008** | **4.64** |
|  | Med (5-8) | -0.030 | -0.97 | 191 | .334 | 0.25 |
|  | High (9-12) | -0.001 | -0.03 | 191 | .980 | 0.16 |
| 5 | Low (1-4) | -0.061 | -1.59 | 191 | .113 | 0.53 |
|  | Med (5-8) | -0.001 | 0.03 | 191 | .980 | 0.16 |
|  | High (9-12) | 0.004 | 0.13 | 191 | .897 | 0.16 |
| 6 | Low (1-4) | -0.05 | -1.31 | 191 | .194 | 0.36 |
|  | Med (5-8) | -0.02 | -0.64 | 191 | .53 | 0.2 |
|  | High (9-12) | -0.002 | -0.08 | 191 | .935 | 0.16 |
| 7 | Low (1-4) | 0.035 | 0.88 | 191 | .379 | 0.23 |
|  | Med (5-8) | -0.001 | -0.04 | 191 | .966 | 0.16 |
|  | High (9-12) | -0.007 | -0.22 | 191 | .827 | 0.17 |
| 8 | Low (1-4) | 0.006 | 0.177 | 191 | .86 | 0.17 |
|  | Med (5-8) | -0.021 | -0.69 | 191 | .488 | 0.20 |
|  | High (9-12) | 0.016 | 0.49 | 191 | .622 | 0.18 |
| 9 | Low (1-4) | 0.029 | 0.89 | 191 | .372 | 0.24 |
|  | Med (5-8) | -0.002 | -0.077 | 191 | .938 | 0.16 |
|  | High (9-12) | 0.001 | 0.03 | 191 | .975 | 0.16 |
| 10 | Low (1-4) | 0.044 | 1.36 | 191 | .175 | 0.38 |
|  | Med (5-8) | 0.008 | 0.28 | 191 | .777 | 0.17 |
|  | High (9-12) | -0.025 | -0.82 | 191 | .413 | 0.22 |
| 11 | Low (1-4) | 0.049 | 1.53 | 191 | .128 | 0.48 |
|  | Med (5-8) | 0.030 | 1.12 | 191 | .265 | 0.29 |
|  | **High (9-12)** | **-0.072** | **-2.34** | **191** | **.02** | **2.02** |

*Note.* DF = degrees of freedom. P-values corrected using the Bonferroni method. Values in bold indicate significant differences in recall between experiments. Negative difference values reflect higher recall for Experiment 4 compared to 3; positive difference values reflect higher recall for Experiment 3 compared to 4.
